# LAMP (Loop-mediated isothermal amplification) assay for rapid identification of Varroa mites

**DOI:** 10.1038/s41598-023-38860-w

**Published:** 2023-07-24

**Authors:** Lea Rako, Arati Agarwal, Lixin Eow, John M. K. Roberts, Brendan C. Rodoni, Mark J. Blacket

**Affiliations:** 1 Agriculture Victoria Research, AgriBio - Centre for AgriBioscience, 5 Ring Road, Bundoora, VIC 3083 Australia; 2 Commonwealth Scientific and Industrial Research Organisation, Canberra, ACT 2601 Australia; 3 School of Applied Systems Biology, La Trobe University, Bundoora, VIC 3083 Australia

**Keywords:** PCR-based techniques, Entomology

## Abstract

Varroa mites are serious pests of European honeybees (*Apis mellifera).* For detection of Varroa mite, a new molecular LAMP-based assay has been developed, which retains the body of the mite intact for morphological identification. Six novel Varroa LAMP primers were designed from existing DNA sequences of the COI locus to target *V. destructor* and *V. jacobsoni,* providing the ability to tell them apart from other non-target beehive associated mite and insect species. This LAMP assay is specific in detecting these *Varroa* species and has been tested on specimens originating from multiple countries. It produces amplification of *V. destructor* and *V. jacobsoni* in 16 ± 3.4 min with an anneal derivative of 78 ± 0.5 °C whilst another *Varroa* species,*V. underwoodi,* showed late amplification. A gBlock gene fragment, used here as a positive control has a different anneal derivative of 80 °C. Three non-destructive DNA extraction methods (HotShot, QuickExtract and Xtract) were tested and found to be suitable for use in the field. The LAMP assay was sensitive to very low levels of Varroa DNA, down to 0.24 picogram (~ 1 × 10 copies/µL of Varroa gBlock). This is a new molecular tool for rapid and accurate detection and identification of Varroa mites for pest management, in areas where these mites do not occur.

## Introduction

Varroa mites (*Varroa jacobsoni* Oudemans and *Varroa destructor* Anderson & Trueman) are damaging ectoparasites of the European honeybee (*Apis mellifera*)^1^, an important pollinator species and honey producer worldwide. These mites harm bees when they feed on developing and adult bees but are also vectors of several bee viruses including deformed wing virus (DWV), which in combination are a major cause of bee colony losses^2,3^. Both Varroa species originate from Asia on their native host the Asian honeybee *Apis cerana* and through two separate host shifts have become parasites of *A. mellifera*^4,5^. *Varroa destructor* has spread widely and become a cosmopolitan species except for Australia and several small island populations^6^, whereas *V. jacobsoni* is restricted outside its original range to Papua New Guinea and Fiji^5^. The impact of Varroa mites includes economic and environmental concerns. The speed of spread of the newly introduced mite into novel environments is generally rapid and devastating as experienced by New Zealand where it took only few years for *Varroa* to establish across both the North and South Island, even after measures were imposed to reduce the movement of honeybees^7,8^.

There are four recognised Varroa species, which are all morphologically similar^9^. Anderson and Trueman (2000) used DNA sequences from the Cytochrome c oxidase subunit 1 (COI) mitochondrial DNA region to show that *V. destructor* is a separate species and identified 18 different Varroa haplotypes in the native range. Furthermore, they identified that only two haplotypes of *V. destructor* have become pests of *A. mellifera* worldwide. The Korea haplotype is the most common and the Japan haplotype is less pathogenic and less common^4,10^. More recently the Java haplotype of *V. jacobsoni* host shifted to *A. mellifera* in Papua New Guinea, becoming a new regional threat to honeybees^5^. The two other species, *V. underwoodi* and *V. rindereri* are restricted to their native host in Asia^11–13^.

Varroa mites reproduce inside the brood cells of honeybees. Each mature female will lay 5–6 eggs at regular intervals, with the first egg being a haploid male^14^. The male develops first and 20 h later the oldest daughter moults to adulthood. Neither males nor immature females can survive outside the brood cell, so the females must be fertilised before the bee emerges to propagate further. In countries that have established populations it is relatively easy to recognise adult Varroa mites using a variety of monitoring methods (e.g., alcohol bee wash and sticky traps placed in the base of hives), although identification of the immature stages is more difficult^15^. By extension, Varroa mites can be difficult to quickly identify by personnel unfamiliar with *V. destructor* in the field, such as in Australia, and can be confused with other bee mites and pollen mites^9,16^. Several laboratory molecular diagnostic assays exist for Varroa mites^17–19^, however there is no suitable molecular method currently available for rapid identification of this pest in the field.

In Australia, Varroa mites are high priority biosecurity pests with national surveillance programs involving sentinel beehives at ports of entry for regular monitoring to enable early interception of Varroa mite incursions^20^. Monitoring involves alcohol washes of adult bees and sticky traps placed at the base of the hives, as well as general inspections for mites^20^. Any mites found require further examination and species identification. Morphological mite identification requires skilled entomologists, and can be time consuming, which can cause delays in a biosecurity response. Molecular diagnostic techniques currently available are also often expensive requiring specialized laboratory facilities and trained staff.

An alternative diagnostic approach is loop-mediated isothermal amplification (LAMP)^21^. Several LAMP assays have been developed for diagnosing plant pests in the field including Queensland fruit fly *Bactrocera tryoni*^22^, grape phylloxera *Daktulosphaira vitifoliae*^23^, Khapra beetle *Trogoderma granarium*^24^ and Fall armyworm *Spodoptera frugiperda*^25^. LAMP provides a new triage tool which can be used to quickly screen and detect pests, with positive detections still able to be confirmed through other methods, such as expert morphological examination or DNA barcoding, if required. In-order to provide rapid and accurate identification of the mites found during inspection of sentinel hives or other interceptions, LAMP can be used as a quick diagnostic tool both in the laboratory and in the field. Reliable positive controls for use in LAMP assays can be synthesised as gBlock Gene Fragments. The gBlock, is a targeted synthetic oligonucleotide and often used as standards in qPCR reactions^26^, and as a synthetic positive control in LAMP reactions to monitor assay performance^24^.

The primary aims of the current study were to: (1) develop and optimise a new molecular diagnostic LAMP assay to provide a rapid and reliable test for identification of Varroa mites; (2) test non-destructive DNA extraction methods for in field use; (3) design a gBlock gene fragment to be used as synthetic positive control; and (4) test the performance of our new LAMP assay for detection of Varroa mites using multiple commercially available master mixes.

## Results

### Varroa LAMP assay design and optimisation

**Figure 1:**
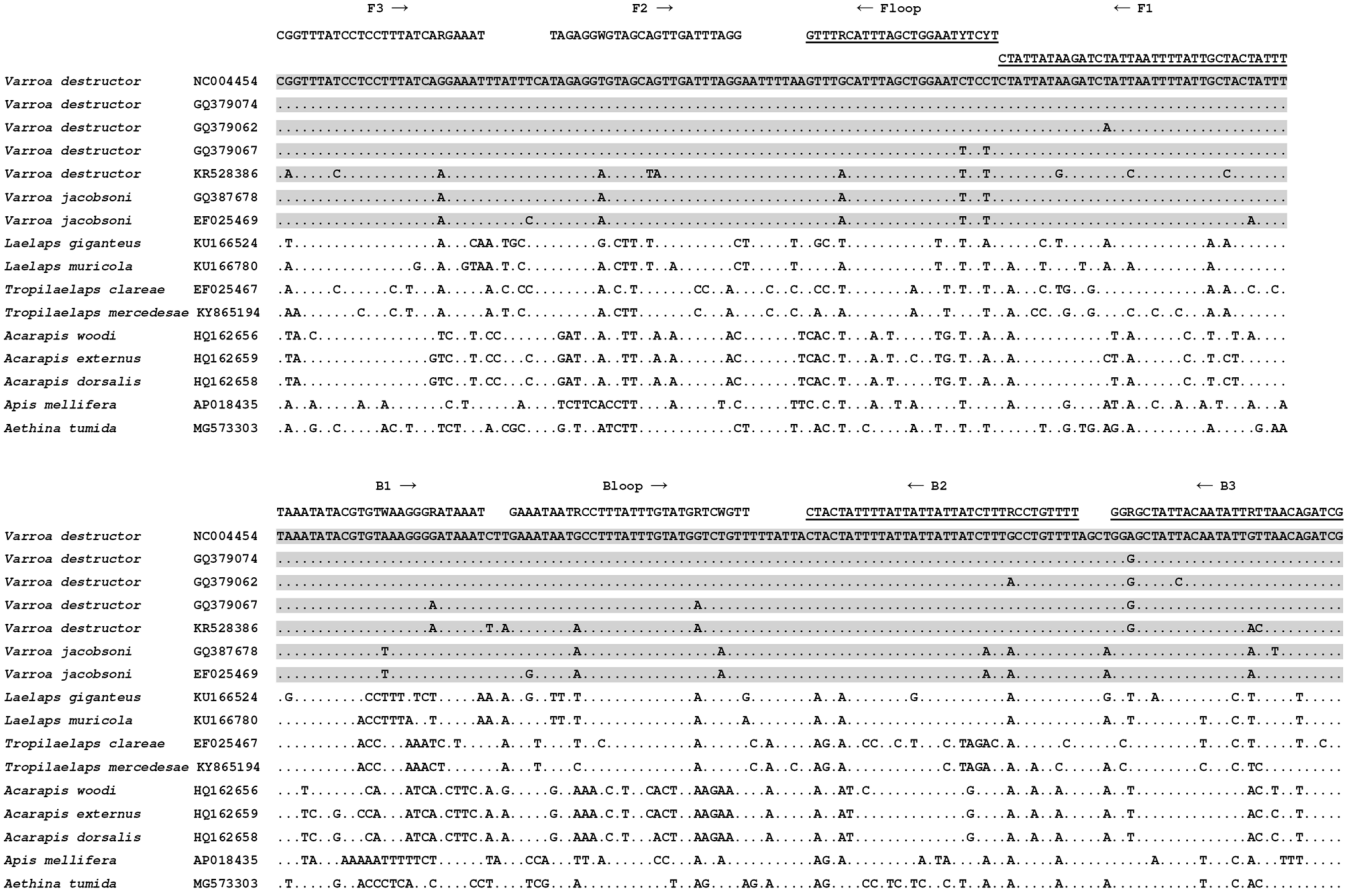
Mitochondrial COI DNA sequence alignment showing Varroa LAMP primer regions. Sequences of *V. destructor* and *V. jacobsoni* (grey shading), and non-target species obtained from GenBank. Reverse primers are underlined; FIP (5’-3’) is made by combining F1 (reverse compliment) and F2; BIP (5’-3’) is made by combining B1 and B2 (reverse compliment).

LAMP primers (Table 1) were developed to target a 259 bp portion of the Varroa COI locus (5’ region) (Fig. 1). Ambiguous bases were added to the primers (Fig. 1, Table 1) to account for common genetic variation present in the wider COI dataset of *V. destructor* and *V. jacobsoni* individuals available on GenBank. Six primers are used in the Varroa LAMP assay, two inner primers (FIP and BIP) and two outer primers (F3 and B3). The addition of loop primers (Floop and Bloop) is beneficial in amplifying positive DNA in less time. The optimised primer ratio (F3/B3: FIP/BIP: Floop/Bloop) was determined to be 1:8:4, with final primer concentrations of 0.4 µM, 3.2 µM and 1.6 µM, respectively.

**Table 1:** Varroa LAMP primer and amplicon sequences (gBlock) and parameters.

LAMP primer or amplicon	Sequence 5'-3'	Primer length (bp)	Predicted Tm (°C)	Primer degeneracy (fold)
Varroa_F3	CGGTTTATCCTCCTTTATCARGAAAT	26	65.1	2
Varroa_B3	CGATCTGTTAAYAATATTGTAATAGCYCC	29	64.0	4
Varroa_FIP	AAATAGTAGCAATAAAATTAATAGATCTTATAATAG**TAGAGGWGTAGCAGTTGATTTAGG**	60	71.4	2
Varroa_BIP	TAAATATACGTGTWAAGGGRATAAAT**AAACAGGYAAAGATAATAATAATAAAATAGTAG**	59	70.3	8
Varroa_Floop	ARGARATTCCAGCTAAATGYAAAC	24	62.2	8
Varroa_Bloop	GAAATAATRCCTTTATTTGTATGRTCWGTT	30	62.8	8
Varroa_gBlock fragment	cccCGGTTTATCCTCCTTTATCAGGAAATcccTAGAGGTGTAGCAGTTGATTTAGGcccGTTTGCATTTAGCTGGAATCTCCTcccCTATTATAAGATCTATTAATTTTATTGCTACTATTTcccTAAATATACGTGTAAAGGGGATAAATcccGAAATAATGCCTTTATTTGTATGGTCTGTTcccCTACTATTTTATTATTATTATCTTTGCCTGTTTTcccGGAGCTATTACAATATTGTTAACAGATCGccc	256	N/A	

The F2 and B2 primer regions of FIP and BIP are shown in bold and underlined. Lowercase letters in the gBlock indicate extra c’s added between LAMP primer sites to increase the overall Tm of the amplicon.

### Varroa LAMP assay specificity results

**Figure 2:**
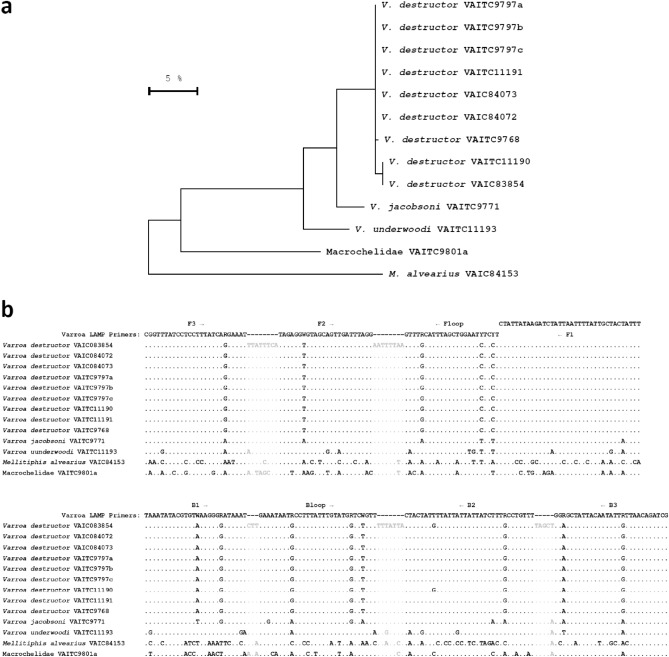
(**a**) Maximum likelihood tree of the mitochondrial COI locus, indicating genetic relationships and sequence variation of mites examined in this study; (**b**) DNA sequence alignment showing DNA sequence variation in the Varroa LAMP assay region. All DNA sequences shown here were generated in this study (GenBank OQ205274–OQ205287).

DNA barcoding confirmed the identification of each *Varroa* species, with 5.3 to 5.8% DNA sequence differences observed between *V. destructor* and *V. jacobsoni*, and 10 to 10.5% difference of either of these species and *V. underwoodi* (Fig. 2). The DNA sequence difference between the *Varroa* species and the non-target mites was > 24% (Fig. 2). The new DNA barcode reference sequences generated in this study have been submitted to GenBank, accession numbers OQ205274—OQ205287 (Table 2).

**Table 2:** Varroa mite specimens tested using the Varroa LAMP assay and 18S LAMP assay.

Species	Origin		Varroa LAMP		18S LAMP		Specimen number	GenBank accession
		DNA extraction method	Time (min)	Temp (°C)	Time (min)	Temp (°C)		
***V. destructor***	**CSIRO (New Zealand)**	**QE, ND**	**18.25**	**77.7**	**24.25**	**88.3**		
***V. destructor***	**CSIRO (Texas-USA)**	**QE, ND**	**14.25**	**77.9**	**20.25**	**88.6**		
***V. destructor***	**NZ PFR (New Zealand)**	**Chelex, D**	**11.75**	**78.5**	**14.75**	**89.1**	VAITC9797b	OQ205274
***V. destructor***	**NZ PFR (New Zealand)**	**Qiagen, ND**	**14.25**	**77.7**	**17.50**	**88.7**		
***V. destructor***	**NZ PFR (New Zealand)**	**Qiagen, ND**	**13.25**	**77.9**	**17.25**	**88.6**	VAITC9768	OQ205275
***V. destructor***	**NZ PFR (New Zealand)**	**Qiagen, D**	**12.50**	**78.2**	**18.75**	**88.7**	VAITC9797c	OQ205276
***V. destructor***	**NZ PFR (New Zealand)**	**HotShot, ND**	**10.75**	**78.2**	**16.50**	**88.8**	VAITC9797a	OQ205277
***V. destructor***	**CSIRO (unknown origin)**	**HotShot, ND**	**11.75**	**78.4**	**18.75**	**89.0**		
***V. destructor***	**CSIRO (unknown origin)**	**HotShot, ND**	**16.25**	**77.9**	**16.25**	**89.1**		
***V. destructor***	**CSIRO (Kuwait)**	**QE, ND**	**23.50**	**77.7**	**15.75**	**88.6**	VAITC11190	OQ205278
***V. destructor***	**CSIRO (unknown origin)**	**QE, ND**	**16.25**	**78.7**	**13.25**	**88.7**		
***V. destructor***	**CSIRO (unknown origin)**	**QE, ND**	**15.75**	**78.5**	**17.75**	**88.2**		
***V. destructor***	**CSIRO (unknown origin)**	**QE, ND**	**16.00**	**78.3**	**13.25**	**88.9**		
***V. destructor***	**CSIRO (unknown origin)**	**QE, ND**	**18.25**	**77.9**	**13.75**	**88.8**	VAITC11191	OQ205279
***V. destructor***	**NZ PFR (New Zealand)**	**Xtract, ND**	**20.00**	**78.1**	**19.75**	**88.3**	VAIC83854	OQ205280
***V. destructor***	**NZ PFR (New Zealand)**	**Xtract, ND**	**15.00**	**78.1**	**14.50**	**88.4**	VAIC84072	OQ205281
***V. destructor***	**NZ PFR (New Zealand)**	**Xtract, ND**	**14.00**	**78.4**	**20.00**	**88.1**	VAIC84073	OQ205282
***V. jacobsoni***	**CSIRO (Papua New Guinea)**	**QE, ND**	**21.50**	**77.3**	**23.75**	**88.5**		
***V. jacobsoni***	**NZ MPI (Fiji)**	**Qiagen, ND**	**20.75**	**77.0**	**19.25**	**88.6**		
***V. jacobsoni***	**NZ MPI (Fiji)**	**Qiagen, ND**	**17.00**	**77.3**	**24.25**	**84.8**	VAITC9771	OQ205283
***V. jacobsoni***	**NZ MPI (Fiji)**	**Qiagen, ND**	**18.50**	**77.3**	**21.75**	**84.7**		
***V. underwoodi***	**CSIRO (PNG)**	**QE, ND**	**24.25**	**76.7**	**24.25**	**84.5**	VAITC11193	OQ205284
*Tropilaelaps mercedesae*	CSIRO (TC-177)	QE, ND	x	x	37.75	84.2		
*Melittiphis alvearius*	AgVic (Victoria)	Qiagen, ND	x	x	24.25	82.8		
*Melittiphis alvearius*	AgVic (Victoria)	Qiagen, ND	x	x	24.00	83.9		
*Melittiphis alvearius*	AgVic (Victoria)	Chelex, ND	x	x	18.00	85.2	VAIC84153	OQ205285
*Acarapis woodi*	NZ MPI (Canada)	Chelex, ND	x	x	x *	x *		
*Acarapis externus*	AgVic (Victoria)	Qiagen, ND	x	x	36.50	88.5		
*Acarapis externus*	AgVic (Victoria)	Qiagen, ND	x	x	39.25	88.5		
Machrochelidae	AgVic (Victoria)	QE, ND	x	x	22.00	83.6	VAITC9801a	OQ205286
Machrochelidae	AgVic (Victoria)	QE, ND	x	x	24.25	84.3	VAITC9801b	OQ205287

*Sample DNA quality of this sample was confirmed through DNA barcoding.

x = No Amplification. Bold indicates *Varroa* species (target taxa). DNA extraction destructive (D), non-destructive (ND), QuickExtract (QE).

**Figure 3:**
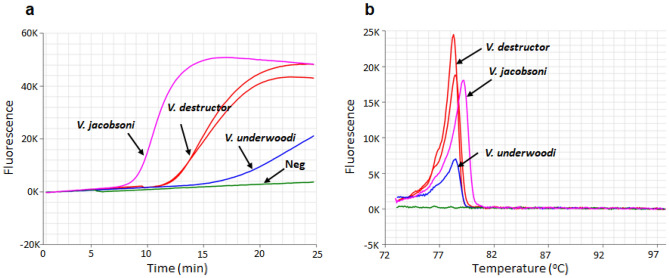
Optimised Varroa LAMP assay results using 1:8:4 (F3/B3: FIP/BIP: Floop/Bloop) primer ratio. (**a**) Amplification profile of three Varroa species: *V. jacobsoni* amplified in 10 min, *V. destructor* in 14 min and *V. underwoodi* in 24 min. Negative, flat line (green). (**b**) anneal derivative temperature for *V. jacobsoni* is 79.2 °C and *V. destructor* and *V. underwoodi* 78.4 °C. Negative, no anneal peak (green).

All 22 specimens of *Varroa* species tested (Table 2) produced positive amplification for the Varroa LAMP assay while the eight non-target mite species did not amplify. The seventeen *V. destructor* specimens amplified in 15.4 ± 3.3 min, while the four *V. jacobsoni* amplified in 19.4 ± 2 min, and the single *V. underwoodi* amplified very late at 24 min (Fig. 3). The anneal derivative temperatures appear very similar between all three Varroa species, at 78 ± 0.5 °C (Fig. 3, Table 2). The 18S LAMP assay confirmed the quality of DNA samples to be high for all the DNA samples, producing positive amplification except for *Acarapis woodi* which failed to amplify for the 18S assay (Table 2). None of the negative non-template controls amplified in any of the LAMP runs performed while optimising the new Varroa assay, confirming the absence of primer dimers and that there was no reagent contamination (Fig. 3).

Different DNA extraction methods, both destructive and non-destructive, were tested using specimens of *V. destructor* (Table 3). All four non-destructive DNA extraction methods yielded good quality and quantity of DNA as assessed with both Varroa and 18S LAMP assays. Three of these non-destructive DNA extraction methods –  HotShot, QuickExtract and Xtract–are suitable for use in the field, as they both yielded *V. destructor* DNA which could be amplified in 12 to 17 min (Table 3). Amplification was quicker using 25 µL of Xtract buffer (14 to 15 min) compared with 50 µL of Xtract buffer, with the larger volume diluting the DNA and increasing the amplification time to 20 min (Table 3).

**Table 3:** Comparison of different DNA extraction methods for *V. destructor* mites for use in Varroa LAMP assay: Destructive (laboratory-based) and Non-destructive (in-field compatible).

DNA extraction methods	n	Varroa LAMP		18S LAMP	
		Time (min)	Temperature (°C)	Time (min)	Temperature (°C)
		Mean ± SD		Mean ± SD	
A. Destructive methods					
Chelex	1	11.8	78.5	14.8	89.1
Qiagen	1	13.0	78.2	18.8	88.7
B. Non-destructive methods					
HotShot	3	12.7 ± 3	78.2 ± 0.3	17.0 ± 1.3	89.0 ± 0.1
Qiagen	2	13.6 ± 0.7	77.8 ± 0.1	17.3 ± 0.1	88.7
QuickExtract	7	17.4 ± 3	78.1 ± 0.4	16.7 ± 4.2	88.6 ± 0.2
Xtract	3	16.3 ± 3	78.2 ± 0.2	18.0 ± 3.0	88.2 ± 0.2

### Varroa LAMP assay sensitivity test

**Figure 4:**
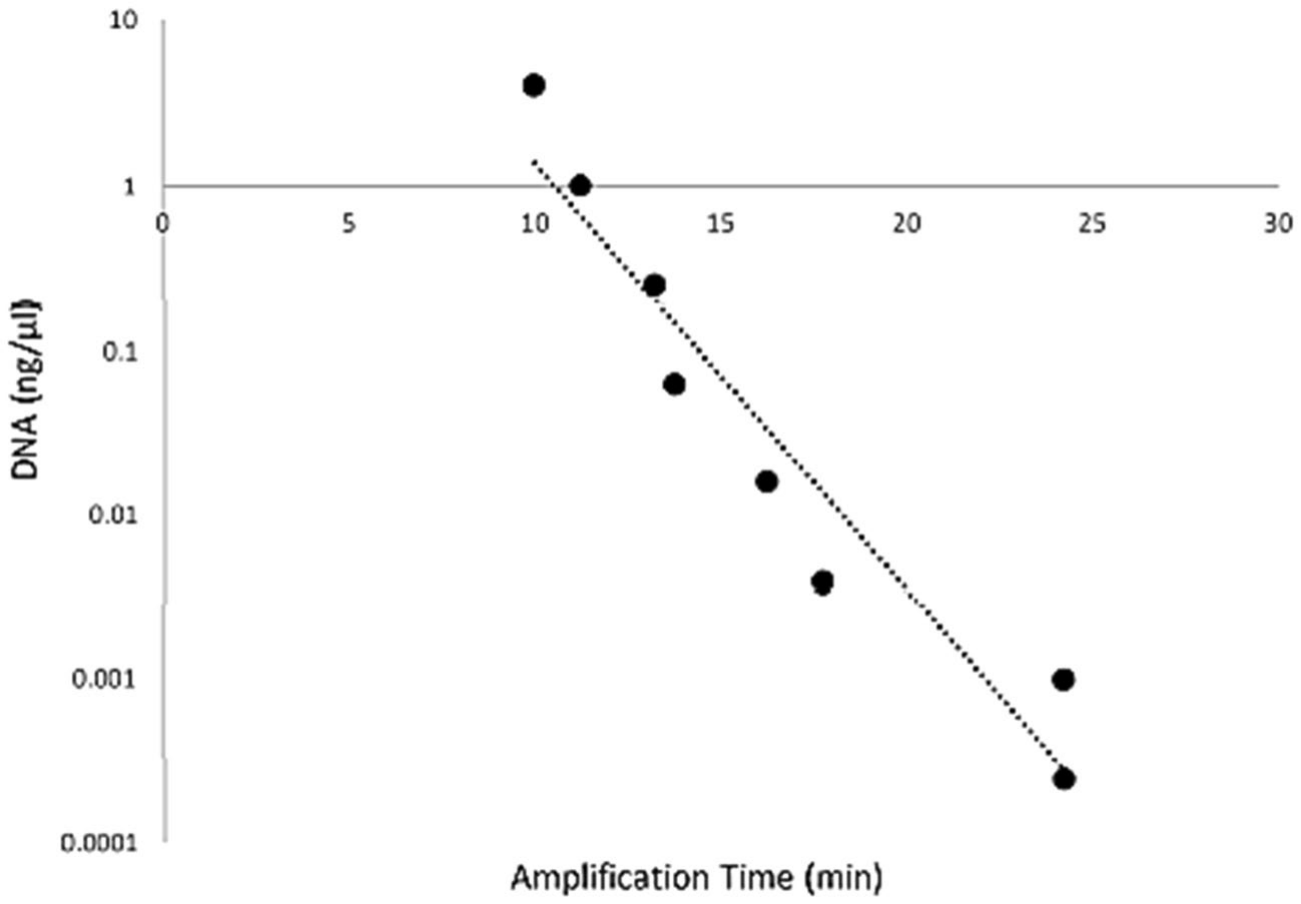
Varroa LAMP sensitivity test using a four-fold DNA dilution series of *V. destructor* (VAITC 9797a). Amplification times are shown for all eight DNA dilutions (black dots) for DNA concentrations ranging from 4.0 ng/µL to 2.44 × 10^–4^ ng/µL. All eight dilutions amplified accordingly from highest DNA concentration in 10 min to lowest DNA concentration in 25 min. Exponential regression line, R^2^ = 0.93.

Sensitivity of the LAMP assay was tested using a four-fold serial dilution of *V. destructor* DNA which produced positive LAMP amplifications for all eight DNA concentrations (Fig. 4). The highest DNA concentration 4 ng/µL produced amplification in 10 min and the lowest DNA concentration 2.44 × 10^–4^ ng/µL amplified in 25 min. The LAMP assay is very sensitive and amplified very low levels of targeted DNA equal to 0.24 picograms of Varroa DNA, with decreasing amounts of DNA resulting in longer amplification times (Fig. 4).

### Evaluation of Varroa gBlock gene fragment

**Figure 5:**
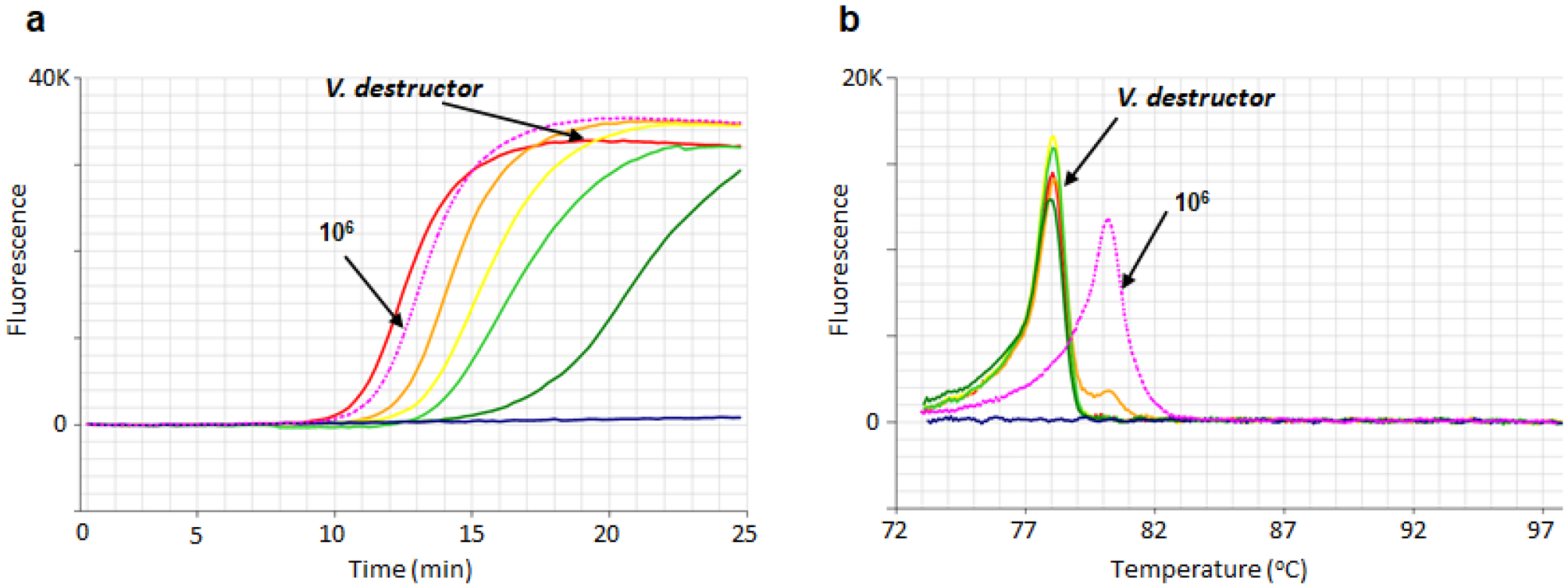
Amplification profile and anneal derivative curve comparison of Varroa gBlock gene fragment (synthetic positive control) and *V. destructor.* **(a)** Amplification profile of five dilutions of *V. destructor* DNA (VAITC 9797a) ranging from 4 ng/µL to 1.56 × 10^–2^ ng/µL (amplification time 10 to 20 min) and gBlock 1 × 10^6^ copies/µL, at 10 min (dotted pink line). Negative, no amplification (blue). **(b)** Anneal derivative of LAMP amplicons showing two peaks, 78 °C for *V. destructor,* and 80 °C for the gBlock (dotted pink line).

The 256 bp Varroa gBlock was very sensitive, being detected as low as ~ 1 × 10 copies/µL of Varroa gBlock within 30 min with an anneal derivative of 80 °C. One million copies (1 × 10^6^) of gBlock amplified within 10 min, which compared to the first dilution of *V. destructor* equating to 4 ng/µL of Varroa DNA (Fig. 5a). Based on this amplification time, 1 × 10^6^ copies/µL of Varroa gBlock was found to be suitable for use as synthetic positive in Varroa LAMP assay. The anneal derivative of LAMP amplicons produced two distinct peaks, 78 °C for Varroa DNA and 80 °C for the gBlock which are easily distinguishable from each other (Fig. 5b).

### Testing the Varroa assay with multiple master mixes

Our optimised Varroa LAMP assay was tested in the laboratory, with the performance of the assay compared using three commercially available master mixes (Table 4). All three *Varroa* species amplified as expected using the three master mixes, with minimal difference in amplification times between them (Table 4). The non-target species did not amplify in all three tests and the two positive control Varroa gBlocks 1 × 10^6^ (from our assay) and 3.8 × 10^8^ (from GWS-K-VDES-02) amplified as expected.

**Table 4:** Testing of Varroa LAMP assay using multiple commercially available isothermal master mixes.

Species	Varroa LAMP (35 min)					
	GWS-K-VDES-08		Isothermal mastermix ISO-001		Isothermal mastermix ISO-004	
	Time (min)	Temp (°C)	Time (min)	Temp (°C)	Time (min)	Temp (°C)
*V. destructor*	11.3	78.6	12.2	77.4	11.0	77.9
*V. jacobsoni*	13.3	78.1	14.2	77.3	12.5	77.9
*V. underwoodi*	24.2	77.8	33.0	77.1	32.2	77.6
*Melittiphis alvearius*	N/A	0.0	N/A	0.0	N/A	0.0
Varroa gBlock 1 × 10^6^	19.0	80.6	14.2	79.9	13.3	80.3
VDES-02 gBlock 3.8 × 10^8^	9.2	80.8	8.5	80.1	7.2	80.4
Neg	N/A	0.0	N/A	0.0	N/A	0.0

## Discussion and conclusion

Rapid response based on a quick and reliable identification of exotic pests is the cornerstone of biosecurity management. Laboratory-based methods for Varroa mite identification are available^4,5^, however species identification is often obtained by engaging trained entomologists, causing time delays in decision making. Additionally, identification of the incomplete specimens, eggs and immature stages is even more difficult, or impossible, by morphological means.

In this study we have developed a simple LAMP assay that reliably identifies Varroa mites. We designed a primer set consisting of six primers, in which F3, B3, FIP and BIP and Loop primers, which target eight regions within the 259 bp section of COI, enable LAMP amplification to be completed in approximately half an hour. When coupled with a rapid DNA extraction method, the complete assay from submitted pest specimen to reliable ‘yes/no’ identification can be performed within one hour. The DNA extraction methods tested in this study, using both laboratory-grade clean, and in-field methods all yielded good quality DNA suitable for LAMP reactions, thus making this assay suitable for in-field use. Notably, we found that decreasing the volume of extraction buffer to 25 µL led to quicker detection times, likely due to an increase in DNA concentration from minutely sized Varroa mites.

Our assay was found to amplify all three *Varroa* species tested, including *V. underwoodi*, which was not included in the initial primer design, as there were no 5’-COI DNA barcodes available for this species at the time. Realigning the Varroa LAMP primers including the newly generated *V. underwoodi* DNA barcode reference sequence generated in our study revealed numerous sequence differences between the *Varroa* species examined; however, although *V. underwoodi* is 9.7 to 10% different for the 259 bp assay region, there was notably only a single base difference in the outer F3 and B3 LAMP primers (Fig. 2b), which are the two primers required to initiate the LAMP amplification process. Interestingly, given that all three species amplified with our new assay it suggests that the LAMP assay will likely not be affected by minor intraspecific haplotypic variation in *V. destructor* or *V. jacobsoni.* To further evaluate the specificity of the Varroa LAMP assay, we tested other relevant mite species closely associated with the bee colonies (*Acarapis sp., Tropilaelaps sp.*) including the benign pollen mite—*Melittiphis alvearius,* which due to its approximate size and shape can easily be confused with Varroa species by the untrained eye. All non-target mites did not amplify even when the LAMP run times were extended, providing confidence that this assay quickly and accurately identifies Varroa mites. Notably, we generated the first DNA barcode reference DNA sequences for *Varroa underwoodi* and *Melittiphis alvearius* in our study to assist with future bee pest diagnostics*.*

Because *Varroa* is an exotic pest for Australia, and DNA is often difficult to obtain in large quantities for use as positive controls, we designed and optimised a synthetic dsDNA (gBlock) for use as positive control in the Varroa LAMP assay, as has been used in other LAMP assays^23–25^. This synthetic DNA fragment has been designed with a different annealing temperature profile (~ 2 °C difference) between the Varroa DNA and gBlock producing two discrete annealing peaks for accurate identification. In practice, gBlocks are quite stable providing large amounts of control DNA when compared with DNA extractions from an individual Varroa mite. The use of a stable gBlock with known concentration further allows tracking of the performance of LAMP assays across runs, and to monitor the integrity of the LAMP reagents.

The DNA extraction and LAMP protocols developed here are simple and easy to use. We demonstrated this by training biosecurity personnel in hands-on training workshops, extracting DNA from mites of *V. destructor* and a non-target Machrochelidae species. The latter were used as negative samples since visually they appear superficially morphologically similar to Varroa mites. The workshop participants had previously not conducted LAMP assays and were trained to use the in-field protocol developed here, obtaining the expected results from the samples tested. Additional testing and training were conducted using a commercially produced version of our new assay (GWS-K-VDES-08, GeneWorks, Australia). We initially tested the lyophilised kit and found that it performed comparably. Advantages of having a commercially available version of this assay include providing quality assured complete sets of reagents that are independent of cool-chain storage requirements (i.e. lyophilised), ready for immediate use for the identification of Varroa mites when required. This is currently especially relevant in Australia, which is undergoing large scale surveys and eradication campaigns, following a detection of *V. destructor* in NSW Australia^27^. Our Varroa LAMP assay has been deployed for use in this response to support standard Varroa mite diagnostics^15^ to assist with preventing further spread outside the incursion area.

We further tested our assay on an alternative technologically simpler LAMP platform, using colorimetric LAMP master mix (WarmStart® Colorimetric LAMP 2 × master mix (DNA and RNA), New England Biolabs Inc., Ipswich, MA, USA) using conditions previously employed^24,25^ and found that we obtained positive reactions (colour change to yellow) at 60 min.

The LAMP-based molecular tool developed in this study allows for rapid and accurate identifications of Varroa mites, providing confidence in whether a species is, or is not, present. This method is simple to use and is highly suitable for early diagnosis of these pests, thus providing technical support to the biosecurity teams by enabling rapid decision making and management of Varroa populations and exotic incursions.

## Materials and methods

### Specimens examined

Specimens of exotic Varroa mites were obtained from three sources through three laboratory visits to: New Zealand Ministry for Primary Industries (MPI, Auckland, New Zealand), New Zealand Institute for Plant and Food Research (PFR, Hamilton, New Zealand), and CSIRO (Canberra, Australia). In total, 22 specimens of *Varroa* species – *V. destructor*, *V. jacobsoni* and *V. underwoodi* – originating from six localities around the world were obtained, seventeen of which were *V. destructor*, the primary target pest, tested from four locations (Table 2).

Identification of specimens representing each *Varroa* species and the two non-target mite species—*Melittiphis alvearius* and Machrochelidae sp. (identified morphologically following Halliday 2000)—were confirmed by DNA barcoding a section. (5′-region) of the mitochondrial COI locus following standard laboratory procedures^29,30^, using the universal primers LCO1490 and HCO2198^31^; and/or C1-J-1718^32^ and COI-REVA^33^.

### DNA extraction

We tested five different DNA extraction methods comprising laboratory-based methods, and non-destructive methods for use in the field, and for sample preservation for further morphological analysis.

1) Qiagen DNeasy Blood and Tissue extraction kit: DNA was extracted from single adult Varroa mites using a DNEasy Blood and Tissue extraction kit (Qiagen), following the manufacturers protocol. These samples provided clean DNA preparations for DNA barcoding (species identification) and as positive controls in LAMP assays. We used column extraction method destructively and non-destructively. Both yielded high quality “clean” DNA, whilst the non-destructive method also enabled the preservation of mite specimens as vouchers for morphological examination.

2) Chelex: Chelex®100 (BIO-RAD), is a chelating resin developed for DNA extraction use suitable for PCR. Fifty microlitres of a 5% (w/v) Chelex suspension and molecular grade water, with the addition of 5 μL proteinase K, was prepared. Varroa mites were homogenised using glass beads in a Mixer Mill at 30 Hz for 3 min, then incubated at 45 °C for 30 min followed by 98 °C for 2 min (Total run time = 32 min). Samples were centrifuged for 2 min at 17 000 rpm to ensure resin beads were absent in suspension, prior to using as template DNA.

3) HotShot: In-field DNA extracts were prepared from single adult Varroa mites using a HotShot “HS6”^34^ protocol as published by Agarwal et al*.* (2020).

4) QuickExtract: In-field DNA extracts were prepared from single adult Varroa mites using QuickExtract™ DNA extraction solution 1.0 (Epicentre, USA) following published protocols^22^.

5) Xtract: Additional in-field DNA extracts were prepared from single adult Varroa mites using Xtract (Xt) DNA extraction solution (GeneWorks, Australia), using 50 μL and 25 μL of Xtract buffer, following published protocols^25^.

For infield application, DNA was extracted by Biosecurity personnel in training workshops using the two non-destructive methods, QuickExtract and Xtract solutions, as outlined above. One μL of DNA was used in each LAMP reaction (see below).

### Varroa dataset and primer design

DNA sequences of the COI locus (5’ region) from *V. destructor, V. jacobsoni*^18^ and non-target species (mites: *Laelaps* sp., *Tropilaelaps* sp., *Acarapis* sp.; insects: European honeybee – *Apis mellifera* Linneaus, small hive beetle – *Aethina tumida* Murray) were obtained from GenBank (Fig. 1). The *Varroa* COI regions of homology were identified manually, and this alignment was used to design a Varroa mite specific LAMP assay. Six novel LAMP assay primers were designed in the present study (Fig. 1, Table 1). For all primers the GC content (%), predicted melting temperature (Tm), and potential secondary structure (hairpins or dimers) were analysed using the Integrated DNA Technologies (IDT) online OligoAnalyzer tool (https://sg.idtdna.com/calc/analyzer), using the qPCR parameters.

### Varroa LAMP assay optimisation

The primer ratio (F3/B3: FIP/BIP: Floop/Bloop) for this assay was optimised following published protocols^22^. The optimised primer mix ratio 1:8:4 was prepared by adding the specified amount of each of the six primers (see below). For a 100 μL volume of primer mix 1:8:4 (F3/B3: FIP/BIP: Floop/Bloop) we added: 10 μL each of F3/B3 (10 µM), 8 μL each of FIP/BIP (100 µM), 4 μL each of Floop/Bloop (100 µM) and 56 μL of Ultrapure water (Invitrogen, Australia).

The Varroa LAMP assay was performed following published protocols^24^. All LAMP assays were run in the Genie III LAMP machine at 65 °C for 25 min to 30 min followed by an annealing curve analysis from 98 °C to 73 °C with ramping down at 0.05 °C/s rate. The total run time being approximately 35 min. The 18S LAMP assay followed published protocols^24^, and was used on all DNA extractions in this study to check for the presence and quality of mite DNA.

### Analytic sensitivity of the Varroa LAMP assay

A four-fold serial dilution of *V. destructor* (specimen VAITC 9797a) (non-destructive DNA extraction using HotShot) DNA was prepared using Ultrapure water (Invitrogen, Life Technologies, Australia). The starting DNA concentration was quantified using a Qubit 2.0 Fluorometer (Invitrogen, Life Technologies, Australia) following the manufacturers protocol. The DNA sample was serially diluted up to 8 dilutions, from 4.0 ng/μl to 2.44 × 10^–4^ ng/μL (1:1 to 1:16,384) and used as template for the Varroa LAMP assay.

### Evaluation of a gBlock gene fragment for Varroa LAMP assay

A gBlock dsDNA fragment (Integrated DNA Technologies, Iowa, USA) was designed for use as synthetic DNA positive control for the Varroa LAMP assay. This synthetic fragment consisted solely of concatenated LAMP primers separated by runs of “ccc”, to increase the overall Tm of the fragment (Table 1). To evaluate detection sensitivity, the copy number and a ten-fold serial dilution (1:10) of the gBlock was prepared as outlined in Agarwal (2022). Sensitivity of the gBlock was tested using 1 × 10^8^ copies/µL to 1 × 10 copies/µL of gBlock in the Genie III, following the Varroa LAMP assay conditions mentioned above (with run time being increased from 25 to 35 min). Following this test, another LAMP run was conducted to determine the best dilution for gBlock to be used as a positive control in LAMP assays. The same four-fold serial dilution of Varroa DNA (VAITC 9797a) (4 ng/µL to 0.0039 ng/µL) was used as template to compare amplification time with one million copies (1 × 10^6^ copies/µL) of gBlock.

### Testing the Varroa assay with multiple master mixes

The performance of the new Varroa LAMP assay was tested using two commercially available isothermal master mixes (ISO-001 and ISO-004, OptiGene, UK) and a commercially produced lyophilised kit (GWS-K-VDES-08, Geneworks, Australia). Each LAMP reaction mix was made by adding 24 µL of master mix and 1 µL of template DNA. Each run included one of each Varroa species – *V. destructor*, *V jacobsoni* and *V. underwoodi*, and the non-target species *Melittiphis alvearius,* 1 × 10^6^ copies/µL of Varroa gBlock, 3.8 × 10^8^ copies/µL VDES synthetic positive control (GWS-K-VDES-08, Geneworks, Australia) and a no-template negative control. All LAMP assays were run in the Genie III at 65 °C for 35 min followed by an annealing curve analysis from 98 °C to 73 °C with ramping at 0.05 °C/s and results analysed on the blue channel.

## Data Availability

GenBank, accession numbers OQ205274–OQ205287.
